# Supplementary figures and images for: Association of KRAS Mutation and Gene Pathways in Colorectal Carcinoma: A Transcriptome- and Methylome-Wide Study and Potential Implications for Therapy
Source: Int J Mol Sci. 2024 Jul 25;25(15):8094. doi: 10.3390/ijms25158094 (PMC11311678; doi:10.3390/ijms25158094)

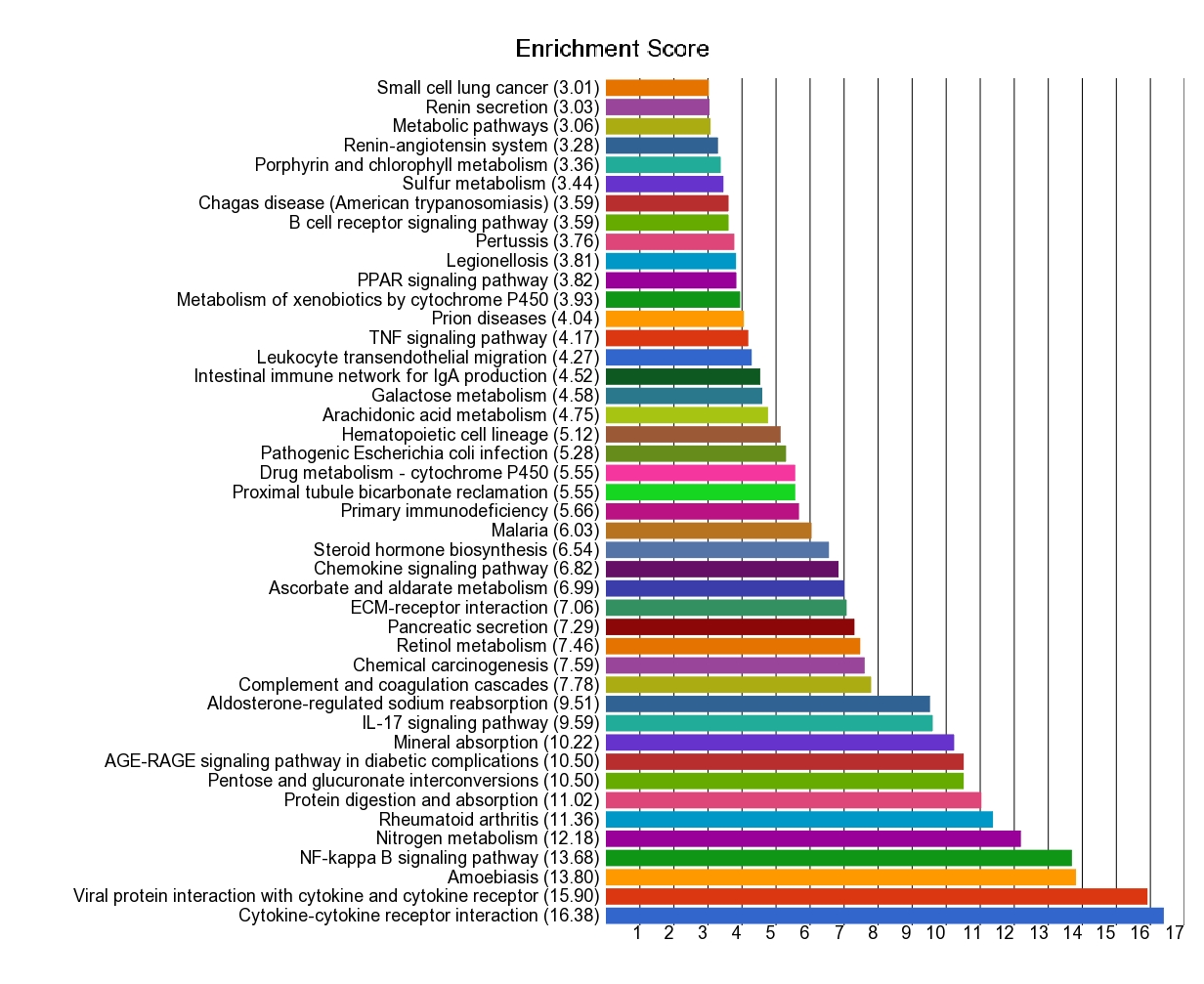

Supplement: Supplementary file 1 [file ijms-25-08094-s001.zip › Figure_S1_Enrichment_FC2FDR0.05ListCombined531_Score_p0.05.jpeg]

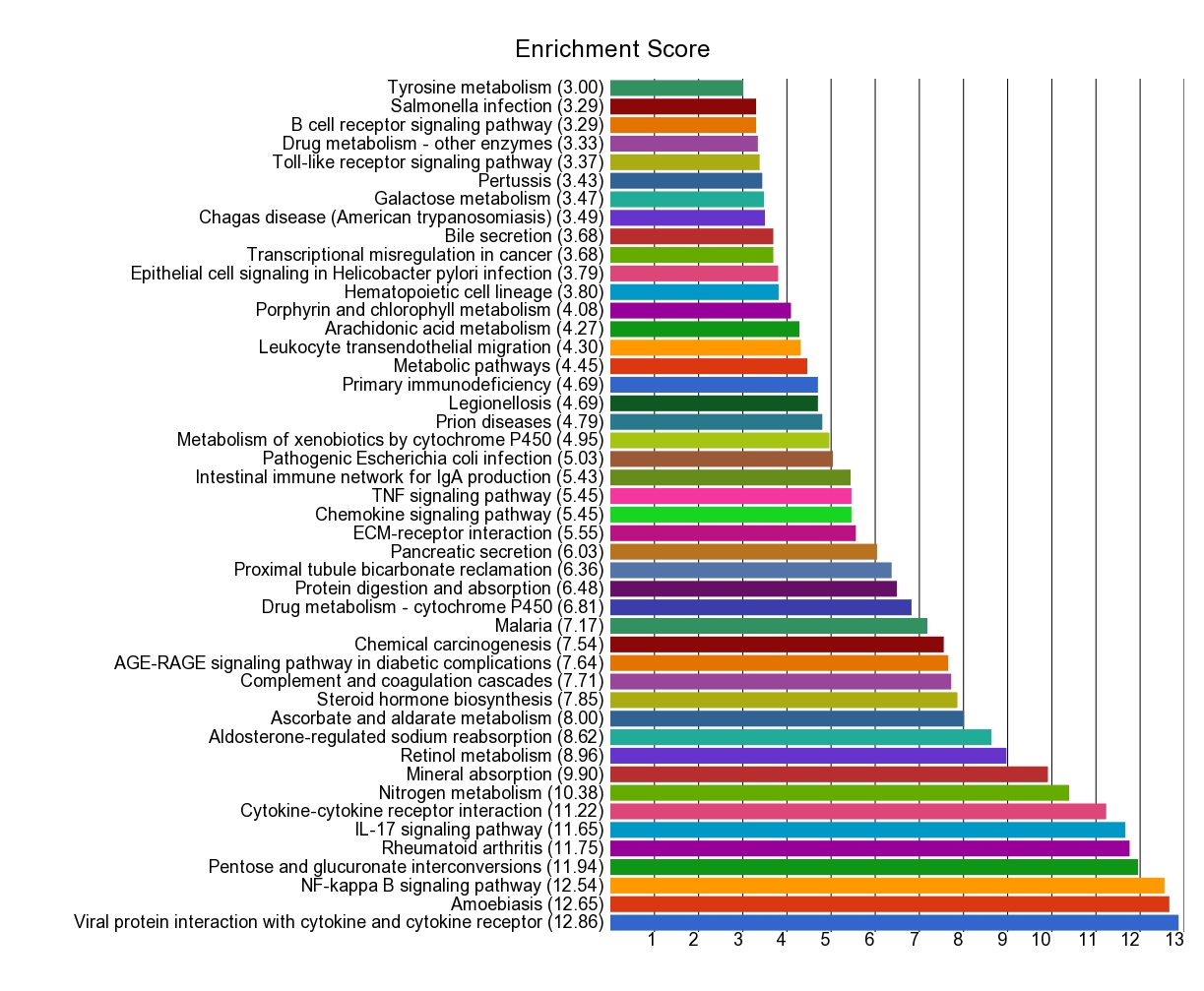

Supplement: Supplementary file 1 [file ijms-25-08094-s001.zip › Figure_S2_Enrichment_FC2FDR0.05Common408_Score_p0.05.jpeg]

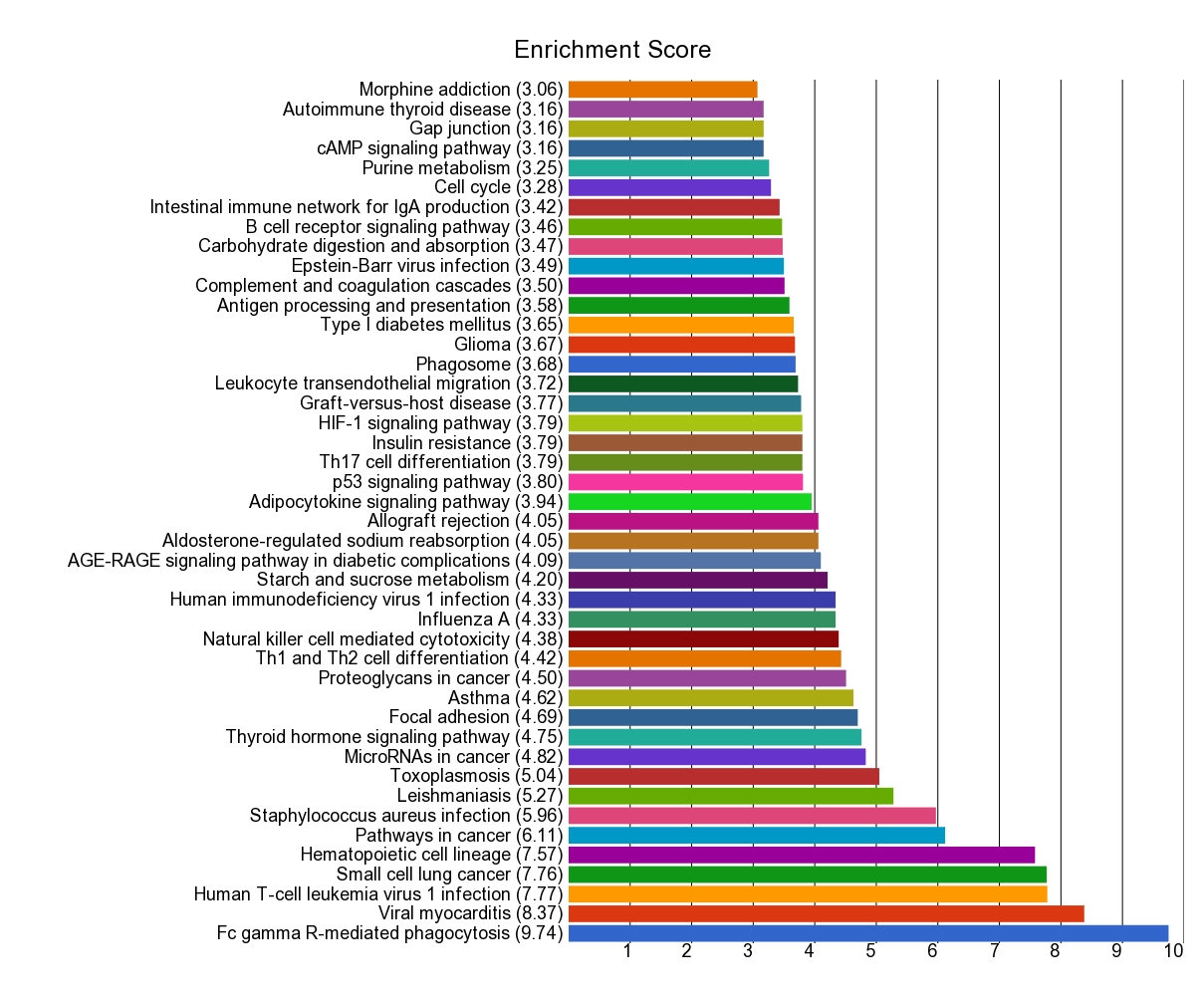

Supplement: Supplementary file 1 [file ijms-25-08094-s001.zip › Figure_S3_Enrichment_FC2FDR0.05KRASmutOnly245_Score_p0.05.jpeg]

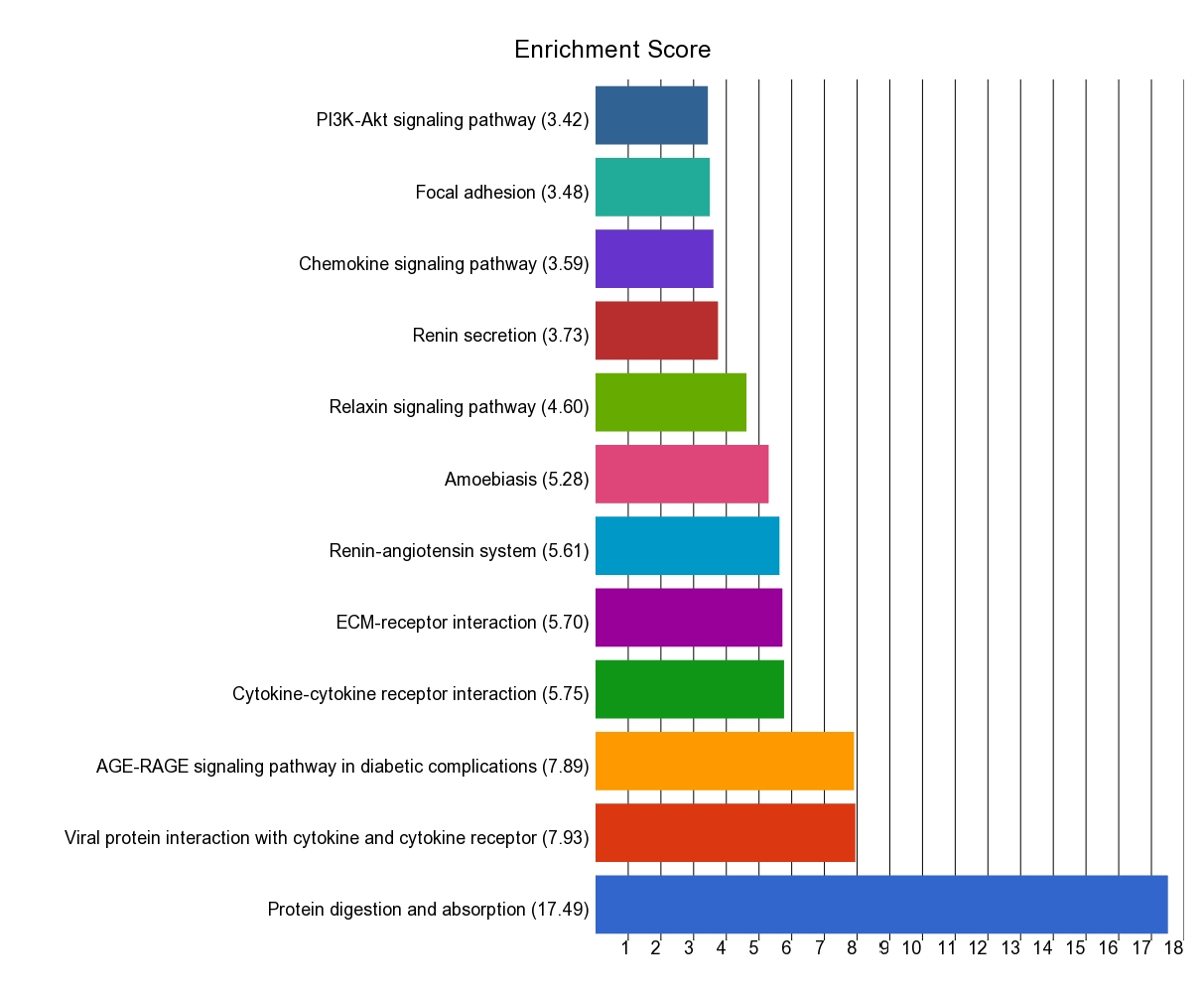

Supplement: Supplementary file 1 [file ijms-25-08094-s001.zip › Figure_S4_Enrichment_FC2FDR0.05KRASwildOnly75_Score_p0.05.jpeg]

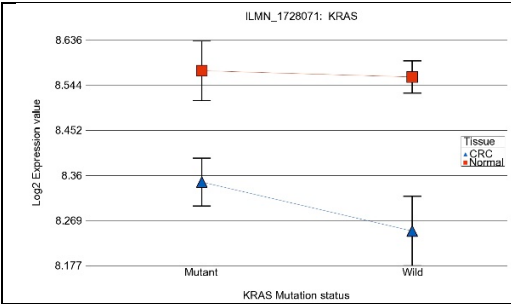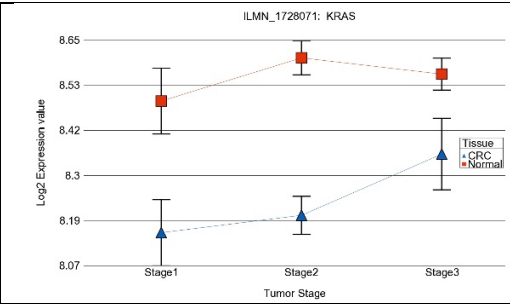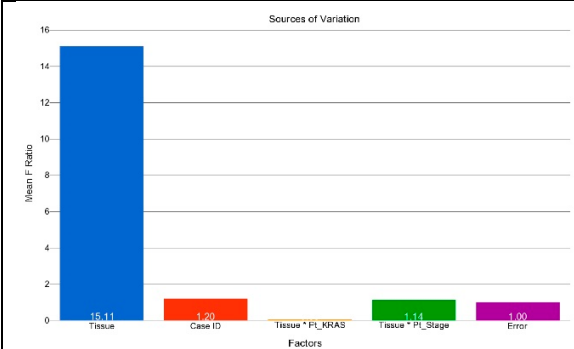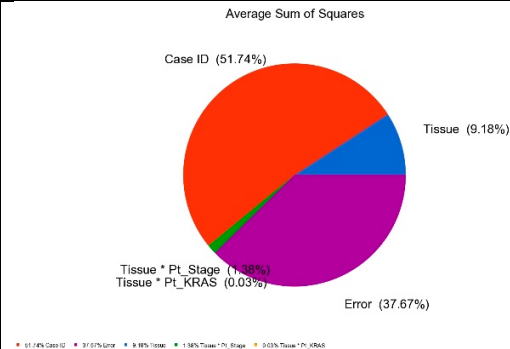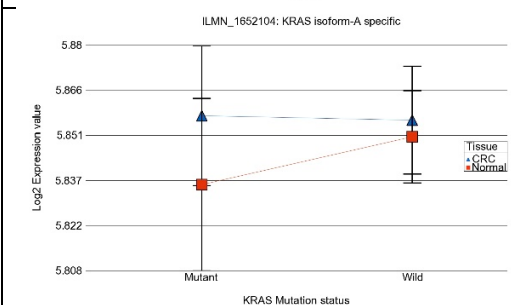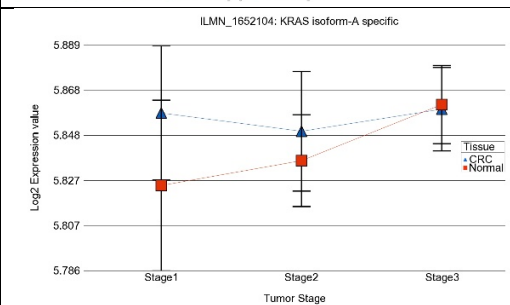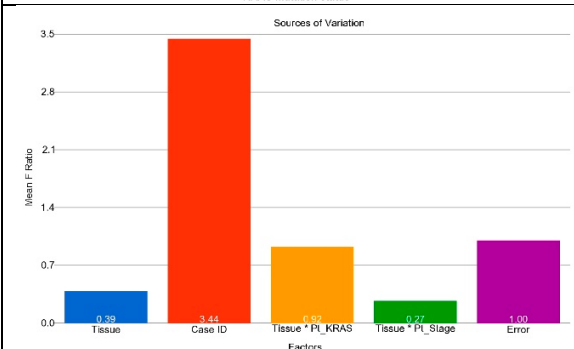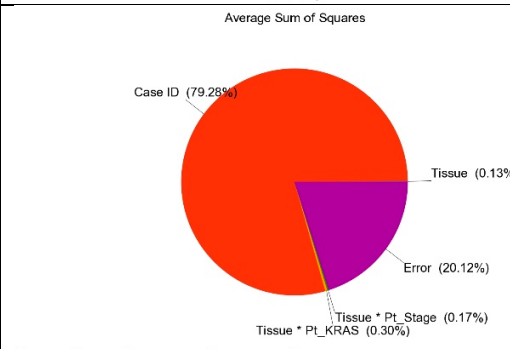

Supplement: Supplementary file 1 [file ijms-25-08094-s001.zip › Figure_S5_Expression of KRAS_by KRAS Mutation status _Stage.pdf]

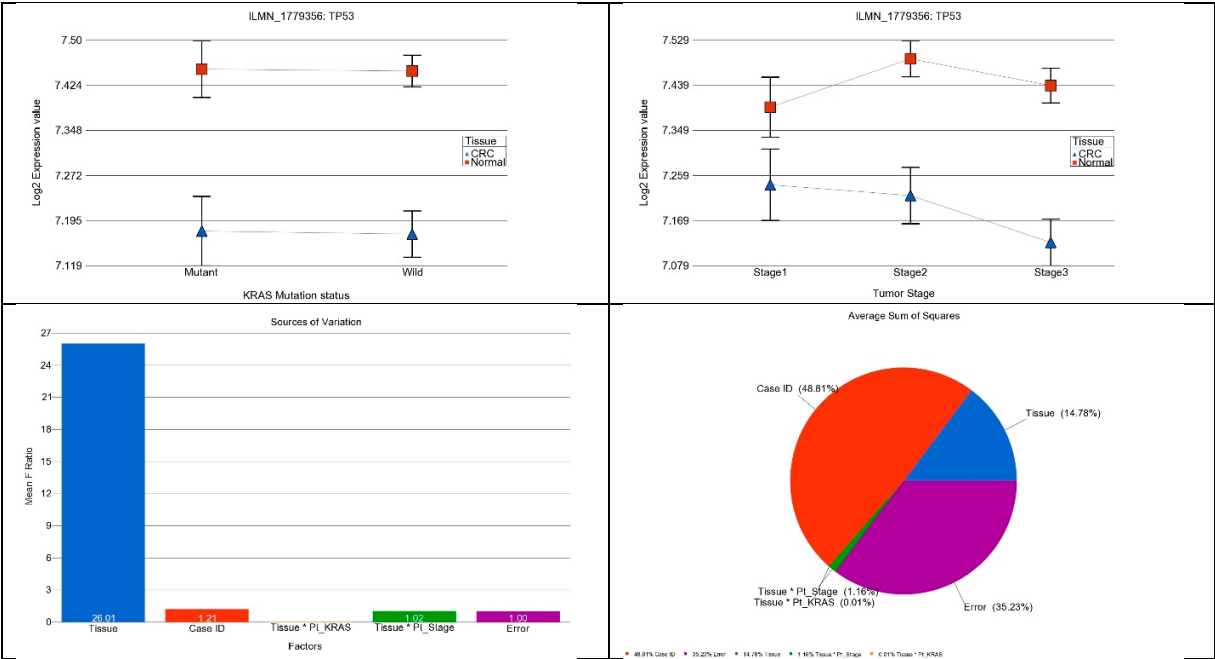

Supplement: Supplementary file 1 [file ijms-25-08094-s001.zip › Figure_S6_Expression of TP53_by KRAS Mutation status _Stage.pdf]
